# A Rough Set Bounded Spatially Constrained Asymmetric Gaussian Mixture Model for Image Segmentation

**DOI:** 10.1371/journal.pone.0168449

**Published:** 2017-01-03

**Authors:** Zexuan Ji, Yubo Huang, Quansen Sun, Guo Cao, Yuhui Zheng

**Affiliations:** 1 School of Computer Science and Engineering, Nanjing University of Science and Technology, Nanjing, Jiangsu, China; 2 School of Computer and Software, Nanjing University of Information Science and technology, Nanjing, Jiangsu, China; Rush University Medical Center, UNITED STATES

## Abstract

Accurate image segmentation is an important issue in image processing, where Gaussian mixture models play an important part and have been proven effective. However, most Gaussian mixture model (GMM) based methods suffer from one or more limitations, such as limited noise robustness, over-smoothness for segmentations, and lack of flexibility to fit data. In order to address these issues, in this paper, we propose a rough set bounded asymmetric Gaussian mixture model with spatial constraint for image segmentation. First, based on our previous work where each cluster is characterized by three automatically determined rough-fuzzy regions, we partition the target image into three rough regions with two adaptively computed thresholds. Second, a new bounded indicator function is proposed to determine the bounded support regions of the observed data. The bounded indicator and posterior probability of a pixel that belongs to each sub-region is estimated with respect to the rough region where the pixel lies. Third, to further reduce over-smoothness for segmentations, two novel prior factors are proposed that incorporate the spatial information among neighborhood pixels, which are constructed based on the prior and posterior probabilities of the within- and between-clusters, and considers the spatial direction. We compare our algorithm to state-of-the-art segmentation approaches in both synthetic and real images to demonstrate the superior performance of the proposed algorithm.

## Introduction

As one of the classical problems in image processing, image segmentation has been extensively studied, which can be treated as a classification problem [1–5] for the target image. Various image segmentation algorithms have been developed such as active contour models [6, 7], graph based methods [8, 9] and clustering techniques [10–12]. Over the last decades, model-based techniques [13, 14] have been widely used in image segmentation, where the standard Gaussian mixture model (GMM) [15, 16] is a well-known method because of its simplicity and ease of implementation [17]. The parameters involved in GMM can be efficiently estimated by expectation maximization (EM) algorithm [18]. However, the standard GMM still suffers from the following limitations: sensitivity to noise, less flexibility to fit the shape of the data and unbounded distributions [19].

In order to reduce noise sensitivity for segmentation, (hidden) Markov random fields ((H)MRF) based mixture models have been widely utilized for pixel labels [20–22], where (H)MRF is acted on neighboring labels, and the clustering result for each pixel depends on the neighboring pixels [23]. On the other hand, in order to impose spatial constraints among neighboring pixels, another group of mixture models with MRF has been proposed by modeling the joint distribution of the priors for each pixel [24–27]. For example, in [26], Displaros et al. proposed a generative GMM model by introducing a pseudo-likelihood quantity to incorporate the spatial smoothness constraints based on Kullback-Leibler (KL) divergence. In [27], Nikou et al. proposed a novel spatial constraint that can adaptively select spatial directions. In order to directly apply the EM algorithm to estimate the involved parameters, Nguyen and Wu [23] proposed a robust spatially constrained GMM by introducing a spatial factor into the prior distribution. Although the forementioned algorithms can reduce the impact of noise in the image, most (H)MRF based algorithms are still not sufficiently robust with respect to different noise types and levels.

Because of the utilization of Gaussian distribution in GMM, the distribution tail is often shorter for many applied problems [17], which means that the Gaussian distribution is not sufficiently flexible to fit data [19]. In order to improve the flexibility for the data fitness, the Student’s-t distribution, Laplace distribution and generalized Gaussian distribution are used to replace the Gaussian distribution in mixture model. Therefore, the Student’s-t mixture model (SMM) [28, 29], Laplace mixture model (LMM) [30, 31], and generalized Gaussian mixture model (GGMM) [32, 33] have been proposed. On the other hand, using only one distribution for each component in the mixture model is not sufficiently satisfactory for many practical applications. Therefore, another solution for fitting data with different shapes is using multiple distributions for each component. For example, Zhang et al. [34] proposed a modified GMM by incorporating local spatial and intensity information. The conditional probability for each pixel is constructed based on the probabilities of neighboring pixels [34]. Browne et al. [35] proposed a mixture of mixture model by combining a multivariate Gaussian distribution and a multivariate uniform distribution together to model the component density [35]. Nguyen et al. [19] proposed an asymmetric mixture model by modeling the component with multivariate Gaussian distributions [19].

Moreover, the distributions in most mixture models are unbounded with a supporting range of (−∞, +∞), which is not consistent with the practical application where the practical data generally fall in a bounded region [19]. In [36], a bounded GMM (BGMM) was proposed for speech processing. In [37], a bounded generalized GMM was proposed that included GMM, LMM, GGMM, and BGMM as special cases. Nguyen et al. [17, 19, 38] proposed various bounded mixture models to fit different data shapes. However, the above mentioned approaches still suffer from the following limitations: (1) Without considering any spatial information, the mixture of mixture model [35] and the bounded asymmetric mixture model (BAMM) [19] are still sensitive to noise, although both of these types of models are more flexible. (2) For the bounded mixture models [19, 36–38], the bounded support regions of observed data should be predefined. Moreover, the indicator function of the bounded support region is a binary function that cannot easily manage uncertainty, vagueness, and incompleteness in data.

Motivated by the aforementioned observations, in this paper, we propose a rough set bounded asymmetric Gaussian mixture model with spatial constraint for image segmentation. First, in our previous work [39], based on the rough set theory [40], we proposed a generalized rough fuzzy c-means (GRFCM) algorithm, where, for each cluster, an image is automatically partitioned into three rough regions with two adaptively computed thresholds. In this paper, we utilize these two thresholds to partition the target image into three rough regions, i.e., the positive, boundary and negative regions [41]. Second, a new bounded indicator function is proposed to determine the bounded support regions of the observed data. The bounded indicator of a pixel that belongs to each sub-region is estimated with respect to the rough region where the pixel lies. Only those pixels in the positive and boundary regions have non-zero indicators. Therefore, because of the benefits of rough set theory, the proposed bounded indicator function can further manage uncertainty in data. Third, to further overcome the impact of noise and reduce over-smoothness for segmentations, two novel prior factors are proposed to introduce the spatial information. The proposed prior factors can be treated as the within- and between-cluster spatial constraints with spatial direction. Finally, to further improve the robustness of the model, for each component, the posterior probabilities of within- and between-cluster for each pixel are estimated with respect to the rough regions. The proposed algorithm is compared to several state-of-the-art segmentation algorithms on simulated and real images to demonstrate its superior performance.

## Finite Mixture Model

The notations used throughout this paper are as follows. The target image is denoted as *X* = {*x*_*i*_, *i* = 1, 2, …, *N*}, where *x*_*i*_ with dimension *D* is the intensity values for the *i*th pixel. The neighborhood of the *i*th pixel is denoted as ∂_*i*_, and the labels are denoted as (Ω_1_, Ω_2_, …, Ω_*K*_). In order to segment an image with *N* pixels into *K* labels, the density function of the finite mixture model [42] is given by:

$$\begin{array}{c} p\left(x_{i}|\Pi ,\Theta \right)=\sum_{k=1}^{K}\pi_{ik}p\left(x_{i}|\Omega_{k}\right), \end{array}$$ (1)

where Π = {*π*_*ik*_}, *i* = {1, 2, …, *N*}, *k* = {1, 2, …, *K*} are the prior probabilities, and satisfy the constraints 0 ≤ *π*_*ik*_ ≤ 1 and $\sum_{k=1}^{K}\pi_{ik}=1$.

In GMM [15, 16], the component *p*(*x*_*i*_|Ω_*k*_) is the Gaussian distribution *Φ*(*x*_*i*_|*μ*_*k*_, Σ_*k*_) that can be written in the form:

$$\begin{array}{c} \Phi \left(x_{i}|\mu_{k},\Sigma_{k}\right)=\frac{exp\{-\frac{1}{2}{\left(x_{i}-\mu_{k}\right)}^{T}\Sigma_{k}^{-1}\left(x_{i}-\mu_{k}\right)\}}{{\left(2\pi \right)}^{D/2}{|\Sigma_{k}|}^{1/2}}, \end{array}$$ (2)

where *μ*_*k*_ is the mean vector with *D* dimension, Σ_*k*_ is the covariance matrix with *D* × *D* dimension, and |Σ_*k*_| is the determinant of Σ_*k*_.

In order to address the issue that the observed data generally fall within the bounded support regions in practical applications, in [19, 36, 37], the bounded support region in ℜ^*D*^ is defined as ∂_Ω_*k*__ for each label Ω_*k*_, and the indicator function can be written as

$$\begin{array}{c} H\left(x_{i}|\Omega_{k}\right)=\{\begin{array}{cc} 1 & \text{if}\;x_{i}\in \partial_{\Omega_{k}}\\ 0 & \text{otherwise} \end{array}. \end{array}$$ (3)

With the above indicator function *H*(*x*_*i*_|Ω_*k*_) and distribution *p*(*x*_*i*_|Ω_*k*_), a bounded distribution $\tilde{p}\left(x_{i}|\Omega_{k}\right)$ can be defined as

$$\begin{array}{c} \tilde{p}\left(x_{i}|\Omega_{k}\right)=\frac{p\left(x_{i}|\Omega_{k}\right)H\left(x_{i}|\Omega_{k}\right)}{\int_{\partial_{\Omega_{k}}}p\left(x|\Omega_{k}\right)dx}. \end{array}$$ (4)

For additional analysis details, please refer to [19, 36] and [37]. However, the major disadvantage of indicator function *H*(*x*_*i*_|Ω_*k*_) is that it is a binary function that cannot easily manage uncertainty in data.

To improve the noise robustness, the spatial information is generally incorporated through MRF distribution:

$$\begin{array}{c} p\left(\Pi \right)=\frac{1}{Z}exp\{-\frac{1}{T}U\left(\Pi \right)\}, \end{array}$$ (5)

where *U*(Π) is the smoothing prior, and *Z* and *T* are two constants. Based on Bayes’rules, the probability density function can be written as:

$$\begin{array}{c} p(\Pi ,\Theta |X)\propto p(X|\Pi ,\Theta )p(\Pi ). \end{array}$$ (6)

Most MRF-based mixture models have been successfully applied to image segmentation by adopting different energy functions *U*(Π). Nguyen and Wu [23] pointed out that the M-step of EM cannot be applied directly to the prior distribution *π*_*ik*_ due to the complexity of the log-likelihood function. Thus, the resulting algorithms are computationally complex and have to utilize large amounts of computational power to solve the constrained optimization problem of the prior distribution *π*_*ik*_ [23]. To overcome these disadvantages, they introduced a novel factor *G*_*ik*_ by defining a multiplication of both posterior probability and prior distributions as follows.

$$\begin{array}{c} G_{ik}=exp[\frac{\beta }{2N_{i}}\sum_{m\in \partial_{i}}\left(z_{mk}+\pi_{mk}\right)], \end{array}$$ (7)

where *z*_*mk*_ is the posterior probability and *β* is the balance parameter to control the smoothing prior. The main advantage of *G*_*ik*_ is the ease of implementation and incorporation of the spatial relationships amongst neighborhood pixels in a simpler metric. Then the smoothing prior *U*(Π) is given by:

$$\begin{array}{c} U\left(\Pi \right)=-\sum_{i=1}^{N}\sum_{k=1}^{K}G_{ik}log\pi_{ik}. \end{array}$$ (8)

However, the energy *U*(Π) can cause over-smoothing for segmentation and loss of details, especially for regions with abundant textures.

## Proposed Model

In order to fit different data shapes, Nguyen et al [19] defined a new distribution *p*(*x*_*i*_|Ω_*k*_) to model the component density. Motivated by the bounded asymmetric distribution, in this paper we modify distribution *p*(*x*_*i*_|Ω_*k*_) to allow the model to easily incorporate the spatial information, which can be defined as:

$$\begin{array}{c} p\left(x_{i}|\Omega_{k}\right)=\sum_{l=1}^{L}\eta_{ikl}\Psi \left(x_{i}|\mu_{kl},\Sigma_{kl}\right), \end{array}$$ (9)

where *L* is the number of bounded multivariate Gaussian distribution *Ψ*(*x*_*i*_|*μ*_*kl*_, Σ_*kl*_), and *η*_*ikl*_ is the weighting factor and satisfies the constraints 0 ≤ *η*_*ikl*_ ≤ 1 and $\sum_{l=1}^{L}\eta_{ikl}=1$. The bounded Gaussian distribution *Ψ*(*x*_*i*_|*μ*_*kl*_, Σ_*kl*_) is defined as:

$$\begin{array}{c} \Psi \left(x_{i}|\mu_{kl},\Sigma_{kl}\right)=\frac{\Phi \left(x_{i}|\mu_{kl},\Sigma_{kl}\right)H\left(x_{i}|\Omega_{k}\right)}{\int_{\partial_{\Omega_{k}}}\Phi \left(x|\mu_{kl},\Sigma_{kl}\right)dx}, \end{array}$$ (10)

where *Φ*(*x*_*i*_|*μ*_*kl*_, Σ_*kl*_) is the Gaussian distribution defined in Eq (2) and *H*(*x*_*i*_|Ω_*k*_) is the indicator function for the bounded support region defined in Eq (3). ∫_∂_Ω_*k*___ *Φ*(*x*|*μ*_*kl*_, Σ_*kl*_)*dx* is the normalization constant.

Therefore, in this paper, we propose a rough set bounded asymmetric Gaussian mixture model with spatial constraint for image segmentation. First, based on the rough set theory, we utilize our previous work [39] to partition the target image into three rough regions with two adaptively computed thresholds. Second, a new bounded indicator function is proposed to determine the bounded support regions of the observed data based on the above rough regions. Third, to further overcome the impact of noise and reduce over-smoothness for segmentations, two novel prior factors with spatial direction are constructed based on the prior and posterior probabilities of the within- and between-clusters. Finally, to further improve model robustness, for each component, the posterior probability is re-estimated based on the adaptively determined rough regions.

### Determination of rough set region

Rough set theory can manage the uncertainty with lower and upper approximations. Specifically, let *U* ≠ ∅ be a universe of discourse, and *R* be an equivalence relationship that can lead to a *U* partition. By denoting *U*/*R* = {*X*_1_, *X*_2_, …, *X*_*n*_}, where *X*_*c*_ is an equivalence class for *R*, the lower and upper approximations of subset *X* are defined as:

$$\begin{array}{c} \left\{ \begin{array}{c} \bar{R}X=\cup \left\{Y\in U/R|Y\cap X\neq \emptyset \right\}\\ \underline{R}X=\cup \left\{Y\in U/R|Y\subseteq X\right\} \end{array}.\right. \end{array}$$ (11)

The lower approximation is a set where all categories certainly belong to *X*, and the upper approximation is a set where all categories possibly belong to *X*. Based on the approximations, three rough regions of *X*, i.e., the R-positive *Po* region, R-negative *Ne* region, and R-boundary *Bo* region, can be defined as follows [41]:

$$\begin{array}{c} Po=\underline{R}X,\;Ne=U-\bar{R}X,\;Bo=\bar{R}X-\underline{R}X. \end{array}$$ (12)

**Fig 1:**
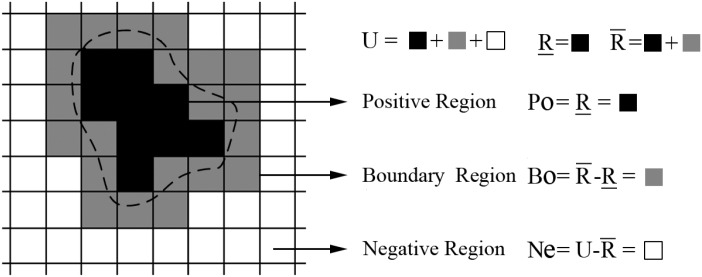
Illustration of three rough regions.

Fig 1 illustrates the definition of the three rough regions. The figure shows that positive region *Po* equals the lower approximation of Ω_*k*_, whereas negative region *Ne* equals the complement of the upper approximation of Ω_*k*_.

In our previous work [39], based on the distance between each pixel value *x*_*i*_ and intensity level *g*_*j*_, two thresholds are adaptively computed to determine the rough regions:

$$\begin{array}{c} d_{i}\left(g_{j}\right)=\frac{\sqrt{\sum_{k\in \partial_{i}}{\left(x_{k}-g_{j}\right)}^{2}}}{n(J_{max}-J_{min})},\;j=1,2,...,J, \end{array}$$ (13)

where ∂_*i*_ is the neighborhood of pixel *i* with *n* pixels. In this paper, the size of neighborhood ∂_*i*_ is set as 3 × 3. *J*_*max*_ and *J*_*min*_ are the maximum and minimum intensity values of the image, respectively, and *J* is the number of intensity levels in the image. Thus, we construct distance vector *d*_*i*_ = {*d*_*i*_(*g*_1_), *d*_*i*_(*g*_2_), …, *d*_*i*_(*g*_*J*_)} for each pixel *i*. The two thresholds can be estimated based on mean value *d*_*i*_*mean*_ and minimum value *d*_*i*_*min*_ of vector *d*_*i*_:

$$\begin{array}{c} t_{1}=\frac{1}{N}\sum_{i=1}^{N}d_{i\_min}\;and\;t_{2}=\frac{1}{N}\sum_{i=1}^{N}d_{i\_mean}. \end{array}$$ (14)

Based on the distance between each pixel *i* and the mean value of class *k* using Eq (13), we can determine the rough regions for each cluster Ω_*k*_ as follows:

$$\begin{array}{c} x\in \{\begin{array}{cc} Po_{k} & \text{if}\;d_{i}\left(\mu_{k}\right)\leq t_{1}\\ Bo_{k} & \text{if}\;t_{1}<d_{i}\left(\mu_{k}\right)\leq t_{2}\\ Ne_{k} & \text{otherwise} \end{array}. \end{array}$$ (15)

For more analysis and discussion details, please refer to [39].

### Construction of bounded support region

As mentioned before, the bounded support regions of observed data were predefined in [19, 36–38], and the indicator function of the bounded support region is a binary function that cannot easily manage uncertainty in data. Motivated by the aforementioned observations, in this paper, we propose a new bounded indicator function based on the rough regions. For each label Ω_*k*_, the bounded support region in ℜ^*D*^ is defined as ∂_Ω_*k*__, and the new indicator function can be written as:

$$\begin{array}{c} \tilde{H}\left(x_{i}|\Omega_{k}\right)=\{\begin{array}{cc} 1 & x_{i}\in Po_{k}\\ \frac{t_{2}-d_{i}\left(\mu_{k}\right)}{t_{2}-t_{1}} & x_{i}\in Bo_{k}\\ 0 & x_{i}\in Ne_{k} \end{array}. \end{array}$$ (16)

With the new indicator function $\tilde{H}\left(x_{i}|\Omega_{k}\right)$ and distribution *p*(*x*_*i*_|Ω_*k*_) in Eq (2), a bounded multivariate Gaussian distribution $\tilde{\Psi }\left(x_{i}|\mu_{kl},\Sigma_{kl}\right)$ can be defined as:

$$\begin{array}{c} \tilde{\Psi }\left(x_{i}|\mu_{kl},\Sigma_{kl}\right)=\frac{\Phi \left(x_{i}|\mu_{kl},\Sigma_{kl}\right)\tilde{H}\left(x_{i}|\Omega_{k}\right)}{\int_{\Omega_{k}}\Phi \left(x|\mu_{kl},\Sigma_{kl}\right)\tilde{H}\left(x|\Omega_{k}\right)dx}, \end{array}$$ (17)

where $\int_{\Omega_{k}}\Phi \left(x|\mu_{kl},\Sigma_{kl}\right)\tilde{H}\left(x|\Omega_{k}\right)dx$ is the normalization constant, and it is identified as the share of *Φ*(*x*_*i*_|*μ*_*kl*_, Σ_*kl*_) that belongs to support region ∂_Ω_*k*__ [19].

Similar to BAMM [19], each component density in our model is constructed with multiple bounded asymmetric distribution. The corresponding distribution *p*(*x*_*i*_|Ω_*k*_) is defined as:

$$\begin{array}{c} p\left(x_{i}|\Omega_{k}\right)=\sum_{l=1}^{L}\eta_{ikl}\tilde{\Psi }\left(x_{i}|\mu_{kl},\Sigma_{kl}\right), \end{array}$$ (18)

where *L* is the number of bounded multivariate Gaussian distribution $\tilde{\Psi }\left(x_{i}|\mu_{kl},\Sigma_{kl}\right)$, and *η*_*ikl*_ is the weighting factor that satisfies the constraints 0 ≤ *η*_*ikl*_ ≤ 1 and $\sum_{l=1}^{L}\eta_{ikl}=1$.

It should be noted that the above distribution always satisfies the conditions of the probability density [14]:

$$\begin{array}{c} \left\{ \begin{array}{c} p(x_{i}|\Omega_{k})\geq 0\\ \int_{-\infty }^{+\infty }p\left(x_{i}|\Omega_{k}\right)dx=\int_{-\infty }^{+\infty }\sum_{l=1}^{L}\eta_{ikl}\tilde{\Psi }\left(x_{i}|\mu_{kl},\Sigma_{kl}\right)=1 \end{array}.\right. \end{array}$$ (19)

Therefore, the log-likelihood function of the proposed model can be written as

$$\begin{array}{ccc} & & L\left(\Pi ,\Theta |X\right)=\sum_{i=1}^{N}\log(\sum_{k=1}^{K}\pi_{ik}p\left(x_{i}|\Omega_{k}\right))\\ & & \;=\sum_{i=1}^{N}\log(\sum_{k=1}^{K}\pi_{ik}\sum_{l=1}^{L}\eta_{ikl}\frac{\Phi \left(x_{i}|\mu_{kl},\Sigma_{kl}\right)\tilde{H}\left(x_{i}|\Omega_{k}\right)}{\int_{\Omega_{k}}\Phi \left(x|\mu_{kl},\Sigma_{kl}\right)\tilde{H}\left(x|\Omega_{k}\right)dx}). \end{array}$$ (20)

In order to maximize the above likelihood function, two variables *z*_*ik*_ and *y*_*ikl*_ are defined as follows:

$$\begin{array}{c} z_{ik}=\frac{\pi_{ik}\sum_{l=1}^{L}\eta_{ikl}\tilde{\Psi }\left(x_{i}|\mu_{kl},\Sigma_{kl}\right)}{\sum_{m=1}^{K}[\pi_{im}\sum_{l=1}^{L}\eta_{ikl}\tilde{\Psi }\left(x_{i}|\mu_{ml},\Sigma_{ml}\right)]}, \end{array}$$ (21)

$$\begin{array}{c} y_{ikl}=\frac{\eta_{ikl}\tilde{\Psi }\left(x_{i}|\mu_{kl},\Sigma_{kl}\right)}{\sum_{m=1}^{L}\eta_{ikm}\tilde{\Psi }\left(x_{i}|\mu_{km},\Sigma_{km}\right)}. \end{array}$$ (22)

The values *z*_*ik*_ and *y*_*ikl*_ always satisfy the conditions $\sum_{k=1}^{K}z_{ik}=1$ and $\sum_{l=1}^{L}y_{ikl}=1$, respectively. It is worth mentioning that, based on Bayes’rules, we can treat both variables *z*_*ik*_ and *y*_*ikl*_ as the posterior probability. Variable *z*_*ik*_ indicates the relationship between pixels and clusters that can be treated as the between-cluster relationship. Meanwhile, variable *y*_*ikl*_ indicates the relationship between pixels and distribution components that can be treated as the within-cluster relationship because each cluster is modeled with multiple distributions. Consequently, variable *z*_*ik*_ can be treated as the posterior probabilities of the between-cluster, whereas variable *y*_*ikl*_ can be treated as the posterior probabilities of the within-cluster.

To further improve model robustness, for each component, the posterior probability of each pixel is re-estimated with respect to the rough region where the pixel lies. Thus, the new hidden variables ${\tilde{z}}_{ik}$ and ${\tilde{y}}_{ikl}$ can be rewritten as:

$$\begin{array}{c} {\tilde{z}}_{ik}=\{\begin{array}{ccc} 1 & \text{if} & x_{i}\in Po_{k}\\ z_{ik} & \text{if} & x_{i}\in Bo_{k}\\ 0 & \text{if} & x_{i}\in Ne_{k} \end{array}, \end{array}$$ (23)

$$\begin{array}{c} {\tilde{y}}_{ikl}=\{\begin{array}{ccc} 1 & \text{if} & x_{i}\in Po_{k}\\ y_{ikl} & \text{if} & x_{i}\in Bo_{k}\\ 0 & \text{if} & x_{i}\in Ne_{k} \end{array}. \end{array}$$ (24)

### Construction of prior factor

As we mentioned before, the prior factor *G*_*ik*_ in [23] plays a role as an average filter on both posterior probability and prior distributions for smoothing noisy images, which may cause over smoothing for the segmentation and lose the details especially for the regions with abundant textures. The smoothing prior in [27] can adaptively select spatial directions, which introduces additional training complexity. To reduce the complexity of the smoothing prior and preserve more details for the segmentations, we propose two novel prior factors *E*_*ik*_ and *F*_*ikl*_ that consider spatial direction based on the prior and posterior probabilities of the between- and within-cluster in this paper. Both prior factors are defined as:

$$\begin{array}{c} E_{ik}=\frac{\exp[\sum_{m\in \partial_{i}^{S_{k}^{*}}}\left(z_{mk}+\pi_{mk}\right)/N_{i}^{S_{k}^{*}}]}{\sum_{h=1}^{K}\exp[\sum_{m\in \partial_{i}^{S_{h}^{*}}}\left(z_{mh}+\pi_{mh}\right)/N_{i}^{S_{h}^{*}}]}, \end{array}$$ (25)

$$\begin{array}{c} F_{ikl}=\frac{\exp[\sum_{m\in \partial_{i}^{S_{l}^{*}}}\left(y_{mkl}+\eta_{mkl}\right)/N_{i}^{S_{l}^{*}}]}{\sum_{h=1}^{L}\exp[\sum_{m\in \partial_{i}^{S_{h}^{*}}}\left(y_{mkh}+\eta_{mkh}\right)/N_{i}^{S_{h}^{*}}]}, \end{array}$$ (26)

where $\partial_{i}^{S_{k}^{*}}$ is the neighborhood of pixel *i* at direction $S_{k}^{*}$ for cluster *k*, which contains $N_{i}^{S_{k}^{*}}$ pixels, and $S_{k}^{*}$ is given by:

$$\begin{array}{c} S_{k}^{*}=\arg\min_{s=1}^{S}\sum_{m\in \partial_{i}^{s}}dist\left(x_{m},\mu_{k}\right), \end{array}$$ (27)

where *dist*(*x*_*i*_, *μ*_*k*_) is the Euclidean distance between point *i* and cluster center *μ*_*k*_, and $\partial_{i}^{s}$ is the neighborhood of pixel *i* at direction *s*. In this paper, we set *S* = 4, i.e., four directions (horizontal, vertical and two diagonal) are considered.

**Fig 2:**
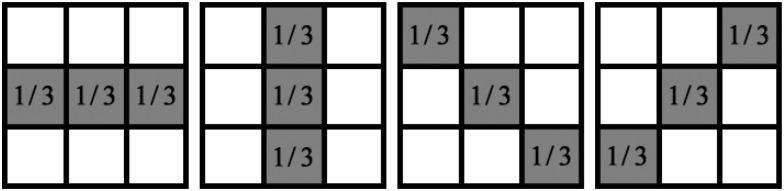
An example of spatial filters that considers four directions. From left to right: horizontal, vertical and two diagonal directions.

More explicitly, taking 3 × 3 neighborhood pixels as example, the filters for each neighboring direction can be constructed as shown in Fig 2. During the algorithm procedure, we only need to operate the convolution over the prior and posterior probabilities with these four predefined filters, and then adaptively select the satisfactory direction based on the differences among the intensity values along each direction. Therefore, the proposed prior factors can efficiently preserve more details.

Then, we incorporate the proposed prior factors into the smoothing prior:

$$\begin{array}{c} U\left(\Pi \right)=-(\sum_{i=1}^{N}\sum_{k=1}^{K}E_{ik}\log\pi_{ik}+\sum_{i=1}^{N}\sum_{k=1}^{K}\sum_{l=1}^{L}F_{ikl}\log\eta_{ikl}) \end{array}$$ (28)

MRF distribution *p*(Π) can be given by:

$$\begin{array}{ccc} & & p\left(\Pi \right)=Z^{-1}\exp\{\frac{1}{T}(\sum_{i=1}^{N}\sum_{k=1}^{K}E_{ik}\log\pi_{ik}\\ & & \;+\sum_{i=1}^{N}\sum_{k=1}^{K}\sum_{l=1}^{L}F_{ikl}\log\eta_{ikl})\}. \end{array}$$ (29)

where *Z* and *T* are set as 1. Therefore, the log-likelihood function of the proposed algorithm can be written in the form:

$$\begin{array}{ccc} L(\Pi ,\Theta |X) & = & \sum_{i=1}^{N}\sum_{k=1}^{K}E_{ik}\log\pi_{ik}+\sum_{i=1}^{N}\sum_{k=1}^{K}\sum_{l=1}^{L}F_{ikl}\log\eta_{ikl}\\ & & +\sum_{i=1}^{N}\log(\sum_{k=1}^{K}\pi_{ik}\sum_{l=1}^{L}\frac{\eta_{ikl}\Phi \left(x_{i}|\mu_{kl},\Sigma_{kl}\right)\tilde{H}\left(x_{i}|\Omega_{k}\right)}{\int_{\Omega_{k}}\Phi \left(x|\mu_{kl},\Sigma_{kl}\right)\tilde{H}\left(x|\Omega_{k}\right)dx}). \end{array}$$ (30)

Finally, we can optimize the involved parameters by maximizing the above log-likelihood function.

### Parameter estimation

In order to determine label Ω_*k*_ for each pixel *x*_*i*_, we need to estimate parameters Π = {*π*_*ik*_, *η*_*ikl*_} and Θ = {*μ*_*kl*_, Σ_*kl*_} by maximizing log-likelihood function *L*(Π, Θ|*X*). By monotonically increasing the logarithm character, maximizing log-likelihood function *L*(Π, Θ|*X*) can lead to minimizing objective function *J*(Π, Θ|*X*) as follows:

$$\begin{array}{ccc} & & J(\Pi ,\Theta |X)=-L(\Pi ,\Theta |X)\\ & & \;=-\{\sum_{i=1}^{N}\log(\sum_{k=1}^{K}\pi_{ik}\sum_{l=1}^{L}\frac{\eta_{ikl}\Phi \left(x_{i}|\mu_{kl},\Sigma_{kl}\right)\tilde{H}\left(x_{i}|\Omega_{k}\right)}{\int_{\Omega_{k}}\Phi \left(x|\mu_{kl},\Sigma_{kl}\right)\tilde{H}\left(x|\Omega_{k}\right)dx})\\ & & \;+\sum_{i=1}^{N}\sum_{k=1}^{K}E_{ik}\log\pi_{ik}+\sum_{i=1}^{N}\sum_{k=1}^{K}\sum_{l=1}^{L}F_{ikl}\log\eta_{ikl}\} \end{array}$$ (31)

By applying the complete data condition in [24], minimizing the negative log-likelihood function in Eq (31) can also lead to minimizing the objective function *E*(Π, Θ|*X*) as follows:

$$\begin{array}{ccc} E(\Pi ,\Theta |X) & = & -\{\sum_{i=1}^{N}\sum_{k=1}^{K}{\tilde{z}}_{ik}[\log\pi_{ik}+\sum_{l=1}^{L}{\tilde{y}}_{ikl}\times \\ & & (\log\eta_{ikl}+\log\tilde{H}\left(x_{i}|\Omega_{k}\right)+\log\Phi \left(x_{i}|\mu_{kl},\Sigma kl\right)\\ & & -\log\int_{\Omega_{k}}\Phi \left(x|\mu_{kl},\Sigma kl\right)\tilde{H}\left(x|\Omega_{k}\right)dx\}])\\ & & -\{\sum_{i=1}^{N}\sum_{k=1}^{K}E_{ik}\log\pi_{ik}+\sum_{i=1}^{N}\sum_{k=1}^{K}\sum_{l=1}^{L}F_{ikl}\log\eta_{ikl}\}. \end{array}$$ (32)

Then we can apply the EM algorithm to minimize Eq (32) by considering the derivation of function *E*(Π, Θ|*X*) with respect to each variable. Finally, we can obtain the following updating function for each variable. Please refer to the Appendix for a detailed derivation of the proposed algorithm.

Mean value estimation:

$$\begin{array}{c} \mu_{kl}=\frac{\sum_{i=1}^{N}{\tilde{z}}_{ik}{\tilde{y}}_{ikl}x_{i}}{\sum_{i=1}^{N}{\tilde{z}}_{ik}{\tilde{y}}_{ikl}}-\frac{\sum_{m=1}^{M}\left(\mu_{kl}-s_{mkl}\right)\tilde{H}\left(s_{mkl}|\Omega_{k}\right)}{\sum_{m=1}^{M}\tilde{H}\left(s_{mkl}|\Omega_{k}\right)}. \end{array}$$ (33)

Covariance matrix estimation:

$$\begin{array}{ccc} \Sigma_{kl} & = & \frac{\sum_{i=1}^{N}{\tilde{z}}_{ik}{\tilde{y}}_{ikl}\left(x_{i}-\mu_{kl}\right){\left(x_{i}-\mu_{kl}\right)}^{T}}{\sum_{i=1}^{N}{\tilde{z}}_{ik}{\tilde{y}}_{ikl}}\\ & & -\frac{\sum_{m=1}^{M}\left(\left(s_{mkl}-\mu_{kl}\right){\left(s_{mkl}-\mu_{kl}\right)}^{T}-\Sigma_{kl}\right)\tilde{H}\left(s_{mkl}|\Omega_{k}\right)}{\sum_{m=1}^{M}\tilde{H}\left(s_{mkl}|\Omega_{k}\right)}. \end{array}$$ (34)

Prior probability estimation:

$$\begin{array}{c} \pi_{ik}=\frac{{\tilde{z}}_{ik}+E_{ik}}{\sum_{h=1}^{K}\left({\tilde{z}}_{ih}+E_{ih}\right)}. \end{array}$$ (35)

$$\begin{array}{c} \eta_{ikl}=\frac{{\tilde{y}}_{ikl}+F_{ikl}}{\sum_{h=1}^{L}\left({\tilde{y}}_{ikh}+F_{ikh}\right)}. \end{array}$$ (36)

Consequently, the proposed algorithm for image segmentation is summarized as follows.

Step 1 Initialize parameters Π = {*π*_*ik*_, *η*_*ikl*_} and Θ = {*μ*_*kl*_, Σ_*kl*_} with the K-means algorithm.
Step 2 Determine the rough regions for each cluster with Eqs (13)–(15).
Step 3 **E-step**
  Update posterior probabilities ${\tilde{z}}_{ik}$ and ${\tilde{y}}_{ikl}$ with Eqs (21)–(24).
Step 4 **M-step**
  Step 4.1 Update prior factors *E*_*ik*_ and *F*_*ikl*_ with Eqs (25) and (26).
  Step 4.2 Update means *μ*_*kl*_ with Eq (33).
  Step 4.3 Update covariance values Σ_*kl*_ with Eq (34).
  Step 4.4 Update prior probabilities *π*_*ik*_ and *η*_*ikl*_ with Eqs (35) and (36).
Step 5 Check the convergence. Stop the iteration if the convergence criterion is satisfied; otherwise, go to Step 2.

It should be noted that the convergence criterion is generally the distance between the values of objective functions or variables (i.e. means or covariance values) from two successive iterations. In this paper, we utilize the total distance between the mean values obtained from two successive iterations. When this distance becomes smaller than a user specified threshold, which was set to 10^−5^ in all algorithms for this study, we think the algorithm converges and stop the iteration.

## Experimental Results

In this paper, we compare the proposed algorithm with four algorithms, i.e., a Bayesian bounded asymmetric mixture model (BAMM) [19], a bounded generalized Gaussian mixture model (BGGMM) [37], a spatially constrained generative model and EM algorithm (SCGM-EM) [26], and a fast and robust spatially constrained GMM (FRSCGMM) [23].

Unless otherwise specified, the parameters of the proposed algorithm are set as follows: The window size for prior factor construction is 3 × 3. The number of bounded multivariate Gaussian distribution is *L* = 3. A summary of the parameter settings for each comparison algorithm is listed in Table 1. Please see the corresponding references for more details. It should be noted that for fair comparison, all algorithms, including the proposed model, use the same initializations generated by the k-means algorithm for each testing image. All algorithms were implemented using MATLAB 7.8 platform and tested on a PC (Intel Core i7-4790 CPU, 3.60GHz, 16GB RAM, and 64-bit Windows 8).

**Table 1:** Summary of parameter setting for each comparison algorithm in experiment.

Algorithms	Parameter setting
SCGM-EM	Temperature value *β* = 0.5
	Size of neighborhood 5 × 5
FRSCGMM	Temperature value *β* = 12
	Size of neighborhood 5 × 5
BAMM	The number of Gaussian distribution *K*_*j*_ = 3
	The number of random variables *M* = 10^6^
	The bounded support regions ∂_Ω_*j*__ ∈ (0, 255)
BGGMM	The number of random variables *M* = 10^6^
	The bounded support regions ∂_Ω_*j*__ ∈ (0, 255)

**Fig 3:**
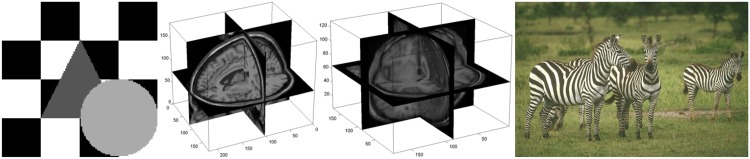
Examples of testing images. From left to right: synthetic image, simulated T1-weighted brain MR, real T1-weighted brain MR and natural images.

The algorithms are compared using synthetic, synthetic and real brain MR, and color images. Fig 3 shows an example of each type of testing image.

(1) The synthetic images used in this paper, of size 128 × 128, contain different labels with luminance values. For example, the image shown at the left of Fig 3 contains four labels with luminance values [0, 1/3, 2/3, 1]. Then we add to the synthetic images different types of noise with different levels for each comparison experiment.

The segmentation results for synthetic images are quantitatively evaluated by correct classification ratio (CCR) [27], which is defined as:

$$\begin{array}{c} CCR=\sum_{k=1}^{K}\frac{|gt_{k}⋂seg_{k}|}{\left|GT\right|}, \end{array}$$ (37)

where *gt*_*k*_ is the ground truth for cluster *k*, *seg*_*k*_ describes the pixels classified by the algorithm to cluster *k* and $GT={⋃}_{k=1}^{K}gt_{k}$. CCR ranges from 0 to 1 with a higher value representing a better segmentation result.

(2) The brain MR images are selected from two open sources: the BrainWeb (http://www.bic.mni.mcgill.ca/brainweb) [43] and IBSRv2.0 (https://www.nitrc.org/projects/ibsr) [44] databases. The objective for brain MR image segmentation is to partition the image into three tissue labels: gray matter (GM), white matter (WM), and cerebrospinal fluid (CSF). The Dice coefficient (DC) [45] is utilized to quantitatively evaluate the performance for segmenting each type of brain tissue, which is the ratio between the intersection and union of segmented volume *S*_1_ and ground truth volume *S*_2_

$$\begin{array}{c} DC\left(S_{1},S_{2}\right)=\frac{2|S_{1}⋂S_{2}|}{|S_{1}\left|+\right|S_{2}|}. \end{array}$$ (38)

The DC value ranges from 0 to 1, with a higher value representing a more accurate segmentation result.

(3) The natural images are selected from the open source Berkeley Segmentation Dataset 500 (BSDS500, https://www2.eecs.berkeley.edu/Research/Projects/CS/vision/bsds/) [46]. The probabilistic rand index (PRI) [47] is used to assess the segmentation performances on natural images. PRI between segmentation map *S*_*seg*_ to be evaluated and a set of *M* ground truth images *S*_*gt*_ = {*S*_1_, …, *S*_*M*_} is given by:

$$\begin{array}{c} PRI\left(S_{seg},S_{gt}\right)=\frac{2}{N(N-1)}\sum_{\begin{array}{c} i,j\\ i<j \end{array}}[c_{ij}p_{ij}+\left(1-c_{ij}\right)\left(1-p_{ij}\right)], \end{array}$$ (39)

where *c*_*ij*_ = 1 if pixels *i* and *j* belong to the same cluster, and *c*_*ij*_ = 0 if pixels *i* and *j* belong to different clusters. PRI takes values between 0 and 1, with a higher value representing a more accurate segmentation result.

### Illustration of proposed algorithm

**Fig 4:**
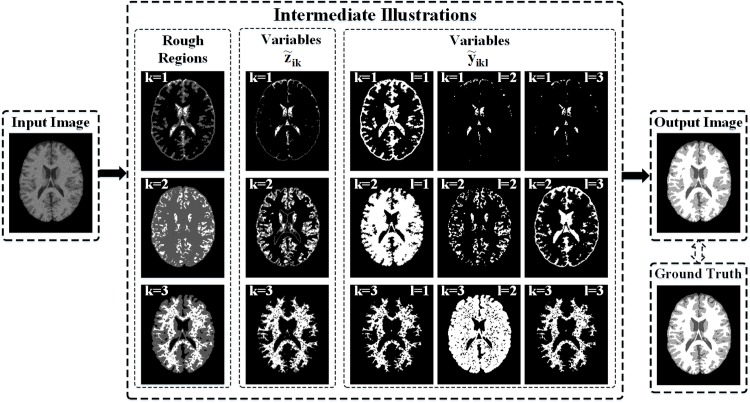
Illustrations of proposed algorithm.

In order to show the advantages and performance of the proposed algorithm in detail, we first segmented a brain MR image that is the 90-th slice of the 3D brain MR image with 9% noise selected from BrainWeb. The component number for the mixture model is *K* = 3. Meanwhile, we set the number of bounded multivariate Gaussian distribution as *L* = 3. The original noisy brain MR image, intermediate illustrations of the proposed algorithm, and final output segmentation are shown in Fig 4. Three rough regions for each cluster, i.e., CSF (*k* = 1), GM (*k* = 2), and WM (*k* = 3) are shown in the first part of the intermediate illustrations, where the positive, boundary, and negative regions are illustrated in bright, gray, and dark respectively. This reveals that the proposed algorithm can construct rough regions appropriately. The corresponding posterior probability of the between and within-cluster, i.e., ${\tilde{z}}_{ik}$ and ${\tilde{y}}_{ikl}$, are depicted in the second and third parts of the intermediate illustrations, respectively. From the nine images of variables ${\tilde{y}}_{ikl}$, we find that variables ${\tilde{y}}_{ikl}$ provide different types of segmentation results for each cluster/tissue, including over and insufficient segmentations, which can further improve the accuracy of the posterior probability estimation by combining with the automatically computed prior probabilities (weights for each distribution). Comparing the last two images on the left of Fig 4, we find that the proposed algorithm can effectively overcome the impact of noise, and the corresponding segmentation result is consistent with the ground truth.

**Fig 5:**
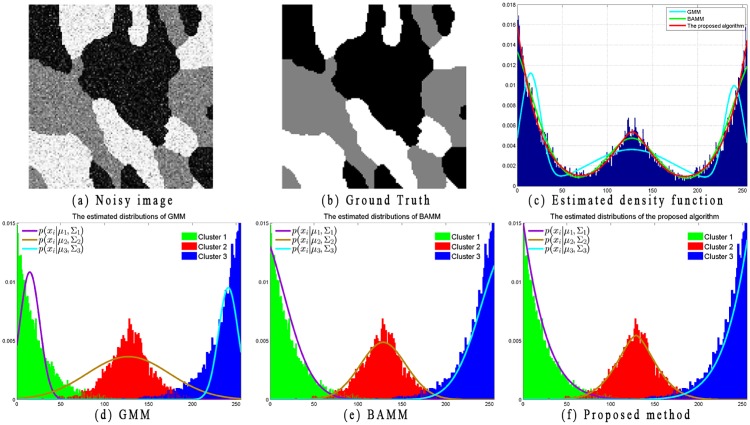
Illustrations of estimated distributions on synthetic image.

**Fig 6:**
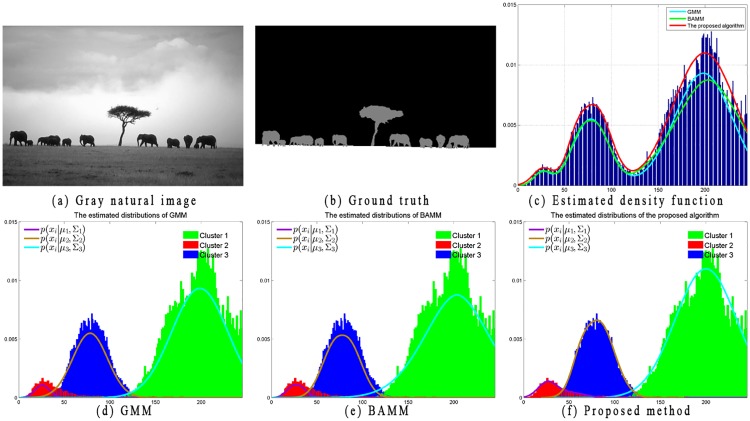
Illustrations of estimated distributions on natural image.

Moreover, to further explain the limitations of GMM and the advantages of the proposed distributions, two examples with image histogram and corresponding estimated distributions are illustrated in Figs 5 and 6. Because BAMM [19] has already proven its superior performance over GMM, SMM, and GGMM, in this section, we only present the estimated distributions obtained by employing *ϕ*(*x*_*i*_|*μ*_*k*_, Σ_*k*_) (i.e., the Gaussian distribution in the GMM model, Eq (2)), *Ψ*(*x*_*i*_|*μ*_*kl*_, Σ_*kl*_) (i.e., the bounded Gaussian distribution in BAMM model, Eq (10)), and $\tilde{\Psi }\left(x_{i}|\mu_{kl},\Sigma_{kl}\right)$ (i.e. the proposed distribution, Eq (17)).

In Fig 5, the noisy image with size 128 × 128 shown in Fig 5(a) is used to compare the performance of the proposed algorithm with GMM and BAMM. This image contains three labels (*K* = 3). The number of bounded multivariate Gaussian distributions for BAMM and the proposed algorithm is set as *L* = 3. The bounded region for BAMM is set as [0, 255] for each cluster. The ground truth of this image is shown in Fig 5(b), which presents the estimated density function obtained by GMM, BAMM, and the proposed algorithm based on the histogram of the observed data. Fig 5(d)–5(f) show the ground truth distributions for each class along with the estimated distributions of each corresponding cluster. From the histogram of observed data and corresponding ground truth distributions, we find that the image data are non-Gaussian, non-symmetric and bounded support data. Without the bounded constraint, GMM performance is not sufficiently satisfactory because the estimated density function cannot fit well with the histogram of observed data. Compared with GMM, both BAMM and the proposed algorithm better fit the observed data because of the introduction of the bounded multivariate Gaussian distribution. It can also be visualized that distribution $\tilde{\Psi }\left(x_{i}|\mu_{kl},\Sigma_{kl}\right)$ in Eq (17) allows flexibility for a better fit of the observed data compared with distribution *Ψ*(*x*_*i*_|*μ*_*kl*_, Σ_*kl*_) of BAMM in Eq (10).

Similar to Figs 5 and 6 shows a gray natural image of size 481 × 321 that is used to compare the performance of the proposed algorithm with GMM and BAMM. This image contains three labels (*K* = 3). In comparison, the estimated distribution of the proposed algorithm is much better than that of the others.

### Segmentation of synthetic images

**Fig 7:**
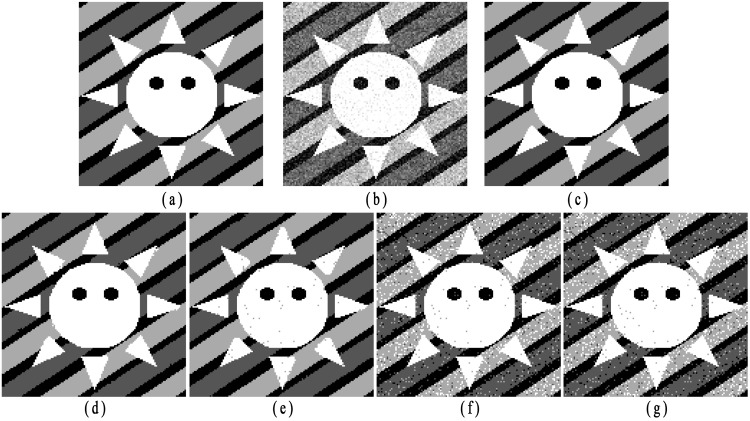
Experimental results on synthetic image with Gaussian noise (image size: 128 × 128). (a) Original image, (b) Noisy image with Gaussian noise (0 mean, 0.07 variance); segmentation results by applying (c) proposed algorithm (CCR = 0.9954), (d) SCGM-EM (CCR = 0.9942), (e) FRSCGMM (CCR = 0.9906), (f) BAMM (CCR = 0.9131), (g) BGGMM (CCR = 0.9152).

In the second experiment, a synthetic image (image size: 128 × 128) as shown in Fig 7(a), is used to compare the performances among different algorithms. The image contains four labels with luminance values [0, 1/3, 2/3, 1]. The noisy image with Gaussian noise (0 means, 0.07 variance) is shown in Fig 7(b). From Fig 7(c)–7(g), we present the segmentation results obtained by employing the proposed algorithm, SCGM-EM, FRSCGMM, BAMM and BGGMM, respectively. Without considering any spatial information, the segmentation accuracies of BAMM and BGGMM are quite poor. The anti-noise ability for FRSCGMM is limited. Both SCGM-EM and the proposed algorithm obtain better performances. Nevertheless, the proposed method obtains higher CCR, especially for the pixels around the boundaries.

**Fig 8:**
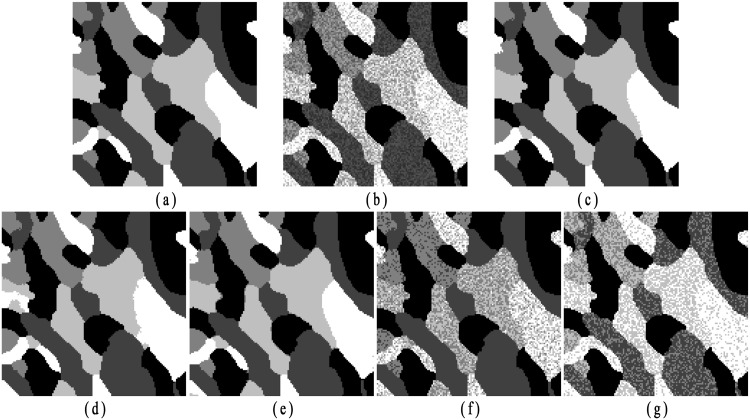
Experimental results on synthetic image with speckle noise (image size: 128 × 128). (a) Original image, (b) Noisy image with speckle noise (0 mean, 0.04 variance), segmentation results by applying (c) proposed algorithm (CCR = 0.9956), (d) SCGM-EM (CCR = 0.9802), (e) FRSCGMM (CCR = 0.9835), (f) BAMM (CCR = 0.8174), (g) BGGMM (CCR = 0.7456).

Then, the synthetic image with luminance values [0, 1/4, 2/4, 3/4, 1] shown in Fig 8(a) is utilized to test the performance of different algorithms on different noise type. As shown in Fig 8(b), the original image is corrupted with multiplicative noise (speckle noise with mean 0 and variance 0.04.). Fig 8(c)–8(g) present the segmentation results obtained by employing the proposed algorithm, SCGM-EM, FRSCGMM, BAMM and BGGMM, respectively. Among these methods, the proposed algorithm obtains better preservation of image details.

### Segmentation of brain MR images

Because of the utilization of our previous work [39], in this section, we supplement the generalized rough fuzzy c-means (GRFCM) algorithm [39] as a comparison method. It should be noted that GRFCM can overcome the impact of intensity inhomogeneity in brain MR images, but all other comparison algorithms can only overcome the impact of noise. Therefore, in this experiment, we apply all algorithms to segment the synthetic T1-weighted 1 mm brain MR images selected from BrainWeb, which only contain different noise levels. For a fair comparison, the intensity inhomogeneity estimation part in GRFCM is removed.

**Fig 9:**
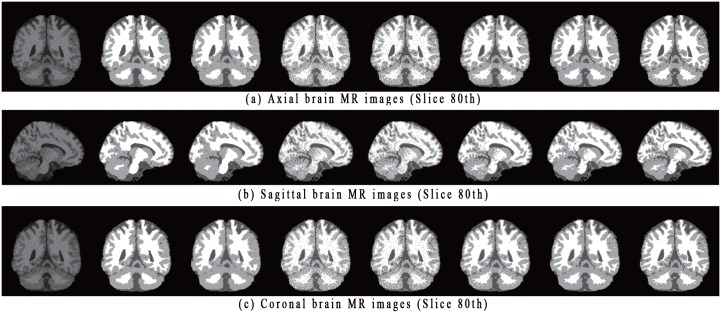
Illustration of three simulated T1-weighted brain MR images with 9% noise and corresponding segmentation results obtained by each algorithm. In each subfigure, the images from left to right show: original image, segmentation results obtained by SCGM-EM, FRSCGMM, BAMM, BGGMM, GRFCM, proposed algorithm, and ground truth.

Three sample brain MR images with 9% noise (80-th axial, sagittal and coronal slice), along with their segmentation results and ground truths, are shown in Fig 9. Similar to the proposed algorithm, both SCGM-EM and FRSCGMM construct spatial information based on the posterior and prior probabilities. However, without considering any directional differences, these two algorithms can obtain over-smoothness segmentations and lose details, especially for the CSF tissue. Meanwhile, both BAMM and BGGMM cannot well distinguish noisy pixels without considering any spatial information. GRFCM is not robust to noise, and the corresponding segmentations are not sufficiently smooth. By comparing the ground truth with the segmentations obtained with all algorithms, we see that the proposed algorithm visually obtains better results.

Segmentation accuracy for each tissue is measured in terms of DC values, and the results are listed in Table 2, which further demonstrates superior performance of the proposed algorithm. It is worth mentioning that the DC values of axial and sagittal CSF segmentation for BAMM and BGGMM are slightly higher than other algorithms because of the low percentage and abundant texture details of the CSF tissue. For any spatially constrained algorithm, the introduced spatial information can improve segmentation accuracy for images occupied by noise, but this can lead to smoothing the texture details in the image. Therefore, it is a dilemma to balance the trade-off between the anti-noise ability and over-smoothness for image textures. By comparing with two other spatially constrained algorithms (SCGM-EM and FRSCGMM), we see that the proposed algorithm achieves better trade-off and balance.

**Table 2:** DC values of each tissue for the segmentations shown in Fig 9.

Algorithms	Tissue	Axial	Sagittal	Coronal	Average
SCGM-EM	GM	0.8854	0.8654	0.9162	0.8890
	WM	0.9090	0.9080	0.9308	0.9159
	CSF	0.8743	0.9032	0.9094	0.8956
FRSCGMM	GM	0.8692	0.8662	0.9075	0.8810
	WM	0.8810	0.9014	0.9137	0.8987
	CSF	0.8417	0.9033	0.9016	0.8822
BAMM	GM	0.8352	0.8268	0.8508	0.8376
	WM	0.8496	0.8522	0.8600	0.8539
	CSF	0.8814	0.9061	0.8707	0.8876
BGGMM	GM	0.8387	0.8241	0.8586	0.8405
	WM	0.8524	0.8554	0.8660	0.8579
	CSF	0.8814	0.9061	0.8921	0.8932
GRFCM	GM	0.8988	0.9135	0.9085	0.9069
	WM	0.8877	0.9140	0.9303	0.9107
	CSF	**0.8853**	**0.9101**	0.8964	0.8962
Proposed algorithm	GM	**0.9106**	**0.8934**	**0.9278**	**0.9106**
	WM	**0.9261**	**0.9235**	**0.9369**	**0.9288**
	CSF	0.8746	0.9040	**0.9126**	**0.8971**

**Fig 10:**
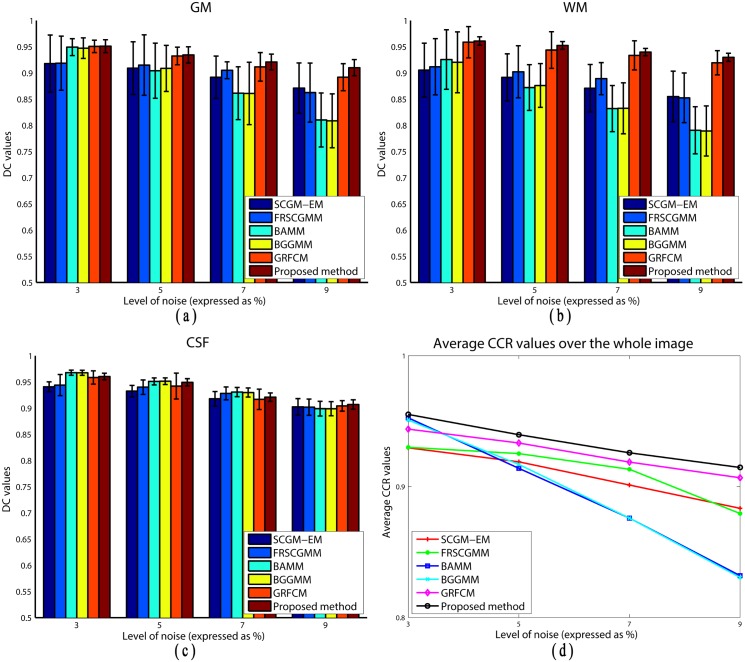
DC values for: (a) GM segmentation, (b) WM segmentation, (c) CSF segmentation, (d) CCR values over the entire images obtained by applying six segmentation algorithms to simulated brain MR images with increasing noise levels.

To statistically show the significance of the proposed algorithm, we apply the previous six algorithms to segment 10 axial, 10 sagittal and 10 coronal (from slice 86th to 95th) MR images for each noise level, where the level ranges from 3% to 9%. The statistical results (means and standard deviations of DC values) are shown in Fig 10(a)–10(c). Moreover, the average CCR value over the entire segmentation results is show in Fig 10(d). From Fig 10, we can observe that the proposed algorithm produces more accurate segmentation (higher means) and has better robustness to noise (lower standard deviations). As indicated before, without considering any spatial information, BAMM and BGGMM produce satisfactory results when the noise level in the image is low (3%). SCGM-EM, FRSCGMM, and the proposed algorithm sacrifice segmentation accuracy for image details in orders to achieve better anti-noise ability. Therefore, the DC values of BAMM and BGGMM for the CSF tissue are slightly higher than the other three algorithms. By increasing the noise level, the performances of BAMM and BGGMM might decrease dramatically, especially for GM and WM tissues. Table 3 lists the statistical results (p-value of paired-sample t-test) between those five methods and the proposed algorithm on the testing images utilized in Fig 10. It is observed that, considering 0.05 as the level of significance, the proposed algorithm provides significantly better segmentation results with respect to both DC and CCR on the BrainWeb dataset.

**Table 3:** Paired-sample t-test results (p-value) of DC and CCR values between the five comparison methods and the proposed algorithm on all the testing images used in Fig 10.

Methods	SCGM-EM	FRSCGMM	BAMM	BGGMM	GRFCM
DC for GM	4.5e-05	2.1e-03	3.8e-20	2.7e-18	1.7e-02
DC for WM	1.9e-06	7.0e-05	2.8e-28	1.0e-25	2.9e-04
DC for CSF	4.1e-04	1.4e-02	1.7e-16	2.8e-15	6.3e-03
CCR	4.5e-06	1.8e-04	4.3e-31	3.9e-28	3.3e-03

In the next experiment, we apply all algorithms on the IBSR v2.0 data set [44], which contains 18 3D images. It is worth mentioning that BAMM [19] has been verified to outperform widely used algorithms, i.e., EMS [45] and SPM [48]. Therefore, in this comparison experiment, we only need to compare BAMM with the proposed algorithm in order to demonstrate the superior performance of the latter.

**Fig 11:**
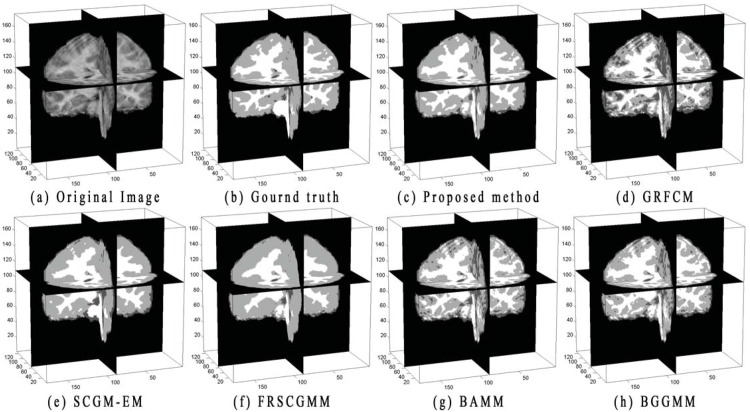
3D slice view of the real dataset (IBSR04), corresponding ground truth and segmentations by applying the proposed method, GRFCM, SCGM-EM, FRSCGMM, BAMM, and BGGMM.

Fig 11 shows a 3D slice view of the real dataset (IBSR04). The image shown in Fig 11(b) is the ground truth of the original image. Fig 11(c)–11(h) show the results obtained by implementing the proposed method, GRFCM, SCGM-EM, FRSCGMM, BAMM, and BGGMM. It is obvious that case IBSR04 contains low contrast between the GM and CSF tissues. BAMM and BGGMM cannot well distinguish the GM and CSF tissues when low contrast occurs. SCGM-EM and FRSCGMM lead to over-smooth segmentations. Without estimation of the intensity inhomogeneity, GRFCM also fails to distinguish the tissues with low contrast. By compareing with these methods, we see that the effect of noise and low contrast on the final segmentation of our algorithm is small and has the most similarity with the ground truth.

**Fig 12:**
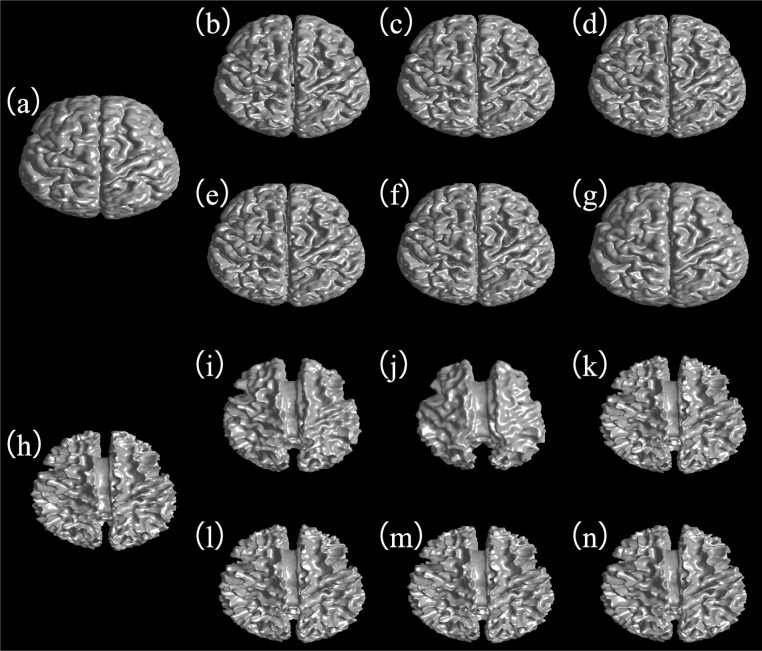
Example of tissue surfaces for case IBSR12. (a) and (h) show ground truth of GM and WM surfaces, respectively. (b) to (g) show GM surface obtained by SCGM-EM, FRSCGMM, BAMM, BGGMM, GRFCM, and the proposed method, respectively. (i) to (n) show the WM surface obtained by SCGM-EM, FRSCGMM, BAMM, BGGMM, GRFCM, and the proposed method, respectively.

Fig 12 shows the tissue surfaces of the segmentation results obtained by six algorithms (SCGM-EM, FRSCGMM, BAMM, BGGMM, GRFCM, and the proposed algorithm) on case IBSR12, and the corresponding ground truth. Fig 12(a) and 12(h) show the ground truth of the GM and WM surfaces, respectively. Fig 12(b)–12(g) show the GM surface obtained by SCGM-EM, FRSCGMM, BAMM, BGGMM, GRFCM, and the proposed method, respectively. Fig 12(i)–12(n) show the WM surface obtained by SCGM-EM, FRSCGMM, BAMM, BGGMM, GRFCM, and the proposed method, respectively. By comparing with the ground truth, it can be observed that our method obtains more accurate segmentation result.

**Fig 13:**
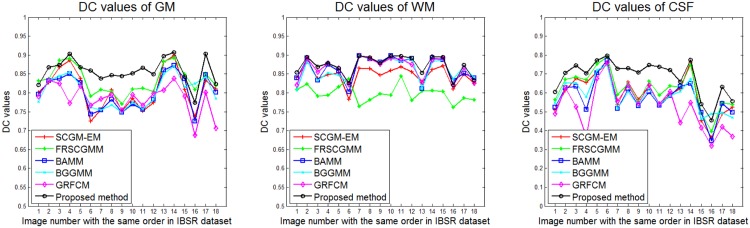
Performance of six segmentation algorithms on 18 benchmark data sets.

Then we tested all those algorithms on 18 cases in the IBSR v2.0 dataset. The segmentation results were assessed in term of DC, and the variation of DC values was depicted in Fig 13. The statistical results, including mean, standard deviation (STD), and p-value of the t-test, of those methods on 18 3D brain images (from IBSR01 to IBSR18) were listed in Table 4. According to the obtained mean and STD, we can tell that the proposed algorithm steadily outperforms other five approaches. Based on the p-values, we find that, considering 0.05 as the level of significance, the proposed algorithm provides significantly more accurate segmentations on the IBSR v2.0 dataset than other five algorithm.

**Table 4:** Statistics of DC values (mean, standard deviation (STD) and p-value) obtained by applying six algorithms to 18 cases from the IBSR v2.0 dataset.

Algorithms	Statistics	GM	WM	CSF
SCGM-EM	Mean	0.8077	0.8470	0.5917
	STD	0.0531	0.0239	0.1051
	p-value	3.6e-05	1.0e-08	9.0e-07
FRSCGMM	Mean	0.8340	0.7986	0.6198
	STD	0.0373	0.0224	0.0975
	p-value	4.2e-03	3.6e-08	1.4e-05
BAMM	Mean	0.8014	0.8644	0.5738
	STD	0.0455	0.0313	0.0952
	p-value	6.6e-07	1.9e-02	3.4e-07
BGGMM	Mean	0.8037	0.8617	0.5826
	STD	0.0531	0.0239	0.1051
	p-value	8.3e-06	1.0e-02	1.8e-06
GRFCM	Mean	0.7849	0.8653	0.5270
	STD	0.0395	0.0292	0.1204
	p-value	6.8e-09	2.4e-03	3.7e-08
Proposed algorithm	Mean	0.8577	0.8662	0.6843
	STD	0.0336	0.0290	0.0925
	p-value	–	–	–

### Segmentation of color images

**Fig 14:**
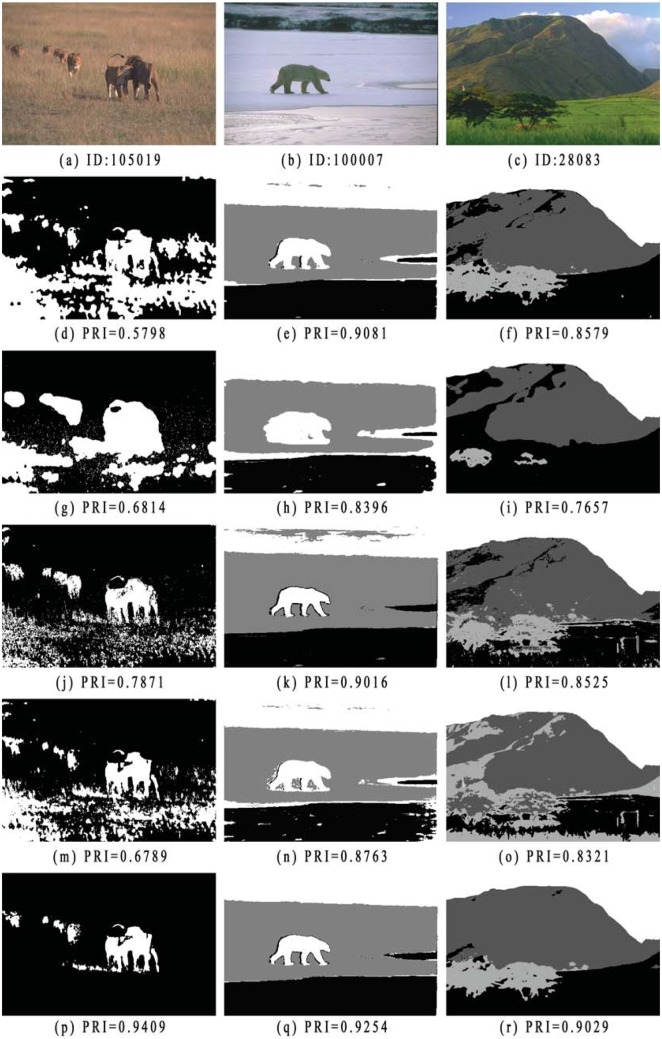
Comparison of color image segmentations. The image IDs are: (a) 105019, (b) 100007, (c) 28083. Images from second to sixth row show segmentation results obtained by SCGM-EM, FRSCGMM, BAMM, BGGMM, and proposed algorithm.

In this section, we test all comparison methods on color images in the Lab color space selected from Berkeley dataset. In Fig 14, we compare the segmentation results of three real-world color images. The first row shows the original images with image ID “105019,”, “100007,” and “28083,” from left to right, and the corresponding number of clusters is 2, 3, and 4. The segmentation results obtained by SCGM-EM, FRSCGMM, BAMM, BGGMM and the proposed SCGAGMM algorithm are shown from the second to sixth row.

The first image (ID: 105019) is segmented into two classes: “lions” and background. As shown in the first row of Fig 14, SCGM-EM, FRSCGMM, BAMM and BGGMM cannot extract the “lions” accurately from the background. Parts of the background region are misclassified as the target. In comparison, the proposed algorithm can successfully extract the target from the background. We attempted to segment the second image (ID: 100007) into three classes, and the proposed method obtains better classification results with more detail. The third image (ID: 374067) consists mainly of four color components: “grassland”, “trees”, “mountain”and “sky”. All comparison methods cannot distinguish well the “trees”or “mountain”from “grassland”. In comparison, our method can successfully segments all objects, especially for the “mountain”region, and does not result in obvious misclassifications.

Finally, a set of color images is tested to evaluate the performance of the proposed algorithm against the SCGM-EM, FRSCGMM, BAMM and BGGMM methods. Table 5 lists the PRI values obtained with all methods for 30 real world images. The statistical results (SR), including mean values, standard deviation (STD), and p-value for the t-test, are listed at the bottom of Table 5. From t-test results, we find that considering 0.05 as the level of significance, the proposed algorithm provides significantly better segmentations with respect to the PRI index. It is evident from the results that the proposed algorithm outperforms other methods with higher PRI values in most cases.

**Table 5:** PRI values of image segmentation results on Berkeley’s color image dataset.

*K*	Image ID	SCGM-EM	FRSCGMM	BAMM	BGGMM	Proposed
2	3063	0.8584	0.8549	0.8372	0.8321	**0.8961**
	3096	0.8770	0.8747	0.7710	0.8720	**0.8779**
	80090	0.7445	0.6368	0.7182	0.7344	**0.7850**
	80099	0.6882	0.8810	0.8041	0.8314	**0.8912**
	105019	0.5798	0.6814	0.7871	0.6789	**0.8789**
	108073	0.6334	0.5603	0.5531	0.6451	**0.7408**
	124084	0.7781	0.7359	0.7467	0.6940	**0.7818**
	135069	0.9746	0.9727	0.9829	0.9829	**0.9841**
	147091	0.8501	0.8340	0.7896	0.7752	**0.8548**
	159091	0.7064	0.7129	0.6962	0.7414	**0.7793**
3	51084	0.6934	0.6834	0.6588	0.6846	**0.6950**
	76002	0.7527	0.7521	0.7667	0.7377	**0.7723**
	113009	0.6683	0.6686	0.6347	0.6108	**0.6725**
	134052	0.5819	0.5657	0.5636	0.5667	**0.7526**
	163014	0.7108	0.7158	0.7188	0.7014	**0.7280**
	176039	0.8345	0.7918	0.8155	0.8057	**0.8393**
	176051	0.7494	0.7431	0.7537	0.6026	**0.7744**
	183055	0.7806	0.7339	0.7843	0.7934	**0.8032**
	249061	0.8847	0.8794	**0.9151**	0.7839	0.9149
	253055	0.9641	0.9086	0.9619	0.7934	**0.9725**
4	14037	0.7634	0.8237	0.8058	0.8039	**0.8245**
	97010	0.8985	0.8940	0.8840	0.8135	**0.9014**
	106025	0.7941	0.7886	0.7943	**0.8340**	0.8223
	117025	0.7836	0.7720	0.7874	0.7852	**0.8112**
	163004	0.7538	0.7111	0.7319	0.7413	**0.7623**
	197017	0.8707	0.8694	0.8487	0.8636	**0.9086**
	198004	0.7587	0.6372	0.7408	0.7210	**0.7922**
	232038	0.8311	0.8125	**0.8382**	0.8030	0.8321
	241004	0.7954	0.7596	0.7923	0.7924	**0.8042**
	361084	0.8090	0.8067	0.8116	0.8002	**0.8273**
SR	Mean	0.7790	0.7687	0.7765	0.7609	**0.8227**
	STD	0.0976	0.1015	0.0970	0.0880	**0.0750**
	p-value	0.0014	2.0e-05	1.4e-05	2.7e-06	–

### Comparison of computational complexity

The computational complexity of those five algorithms was compared in Table 6, where *T* is the number of iterations when the algorithm converges, *N* is number of pixels in an image, *D* is the dimension of each pixel, *K* is the number of clusters, *L* is the number of distributions and *N*_∂_*i*__ is the number of pixels in the neighborhood ∂_*i*_. It should be noted that the computational complexity for the EM algorithm is of the order *O*(*NKD*^2^) for each iteration [49].

**Table 6:** Computational complexity, converging time, number of iterations and per iteration time (average ± standard deviation, UNIT: Second) by applying five algorithms on BrainWeb dataset.

Algorithms	SCGM-EM	FRSCGMM	BAMM	BGGMM	Proposed
Computational complexity	$O({\mathrm{NKD}}^{2}TN_{\partial_{i}}^{2})$	$O({\mathrm{NKD}}^{2}TN_{\partial_{i}}^{2})$	*O*(*NKD*^2^*TL*)	*O*(*NKD*^2^*TL*)	$O({\mathrm{NKD}}^{2}TLN_{\partial_{i}}^{2})$
Converging time	0.3804 ± 0.1090	0.9808 ± 0.3585	3.4747 ± 0.6914	3.4901 ± 1.4738	4.4978 ± 0.6397
Number of iterations	41.25 ± 11.90	109.70 ± 39.58	160.90 ± 25.06	38.40 ± 15.94	37.60 ± 19.29
Per iteration time	0.0092 ± 0.0001	0.0088 ± 0.0001	0.0207 ± 0.0005	0.0780 ± 0.0012	0.1391 ± 0.0519

Table 6 also gives the converging time, number of iterations and time-cost per iteration of five algorithms obtained by applying each of them to 100 2D brain MR images, which have a size of 217 × 181 and were selected from the BrainWeb dataset. In this comparative experiment, we checked for the convergence of the parameter values, set the stopping criteria to *ε* = 10^−5^ and executed each algorithm with 100 iterations (Intel Core i7-4790 CPU, 3.60GHz, 16GB RAM, 64-bit Windows 8, and Matlab Version 7.8). It is worth mentioning that the proposed algorithm was performed in the MATLAB environment without any particular code optimization.

## Conclusion

To overcome the limitations involved in most GMM-based algorithms, in this paper, we proposed a rough set bounded asymmetric Gaussian mixture model with spatial constraint for image segmentation. Based on the rough set theory, a new bounded indicator function was proposed to determine the bounded support regions of the observed data. The bounded indicator and posterior probability of a pixel that belongs to each sub-region were estimated based on the rough regions. The within- and between-cluster spatial constraints were introduced by incorporating the spatial information with adaptively selected direction in order to reduce over-smoothness for segmentations. Experimental results demonstrated that the proposed algorithm is flexible to fit the data shapes, and robust to noise, which makes our method be capable of producing more accurate segmentation results comparing with several state-of-the-art algorithms. Future work will be devoted to reducing the complexity of the proposed algorithm.

## Appendix

### Mean value estimation

Considering the derivation of function *E*(Π, Θ|*X*) in Eq (32) with respect to *μ*_*kl*_, we have

$$\begin{array}{ccc} \frac{\partial E(\Pi ,\Theta |X)}{\partial \mu_{kl}} & = & -\sum_{i=1}^{N}{\tilde{z}}_{ik}{\tilde{y}}_{ikl}\{-\Sigma_{kl}^{-1}\left(\mu_{kl}-x_{i}\right)\\ & & +\Sigma_{kl}^{-1}\frac{\int_{\Omega_{k}}\Phi \left(x|\mu_{kl},\Sigma_{kl}\right)\tilde{H}\left(x|\Omega_{k}\right)\left(\mu_{kl}-x\right)dx}{\int_{\Omega_{k}}\Phi \left(x|\mu_{kl},\Sigma_{kl}\right)\tilde{H}\left(x|\Omega_{k}\right)dx}\}, \end{array}$$ (40)

where the term $\int_{\Omega_{k}}\Phi \left(x|\mu_{kl},\Sigma_{kl}\right)\tilde{H}\left(x|\Omega_{k}\right)\left(\mu_{kl}-x\right)dx$ is the expectation of function $\tilde{H}\left(x|\Omega_{k}\right)\left(\mu_{kl}-x\right)$ under distribution *Φ*(*x*|*μ*_*kl*_, Σ_*kl*_), which can be approximated as [14, 19, 36]:

$$\begin{array}{c} \int_{\Omega_{k}}\Phi \left(x|\mu_{kl},\Sigma_{kl}\right)\tilde{H}\left(x|\Omega_{k}\right)\left(\mu_{kl}-x\right)dx\approx \frac{1}{M}\sum_{m=1}^{M}\left(\mu_{kl}-s_{mkl}\right)\tilde{H}\left(s_{mkl}|\Omega_{k}\right), \end{array}$$ (41)

where *s*_*mkl*_ ∼ *Φ*(*x*|*μ*_*kl*_, Σ_*kl*_) is the random vector drawn from distribution *Φ*(*x*|*μ*_*kl*_, Σ_*kl*_), and *M* is the number of random vectors *s*_*mkl*_ [19]. In this paper, we set *M* = 10^6^ for all experiments.

Similarly, the term $\int_{\Omega_{k}}\Phi \left(x|\mu_{kl},\Sigma_{kl}\right)\tilde{H}\left(x|\Omega_{k}\right)dx$ can be approximated as [14, 19, 36]:

$$\begin{array}{c} \int_{\Omega_{k}}\Phi \left(x|\mu_{kl},\Sigma_{kl}\right)\tilde{H}\left(x|\Omega_{k}\right)dx\approx \frac{1}{M}\sum_{m=1}^{M}\tilde{H}\left(s_{mkl}|\Omega_{k}\right). \end{array}$$ (42)

Based on Eqs (41) and (42), ∂*E*(Π, Θ|*X*)/∂*μ*_*kl*_ from Eq (40) can be rewritten as:

$$\begin{array}{ccc} \frac{\partial E(\Pi ,\Theta |X)}{\partial \mu_{kl}} & = & -\sum_{i=1}^{N}{\tilde{z}}_{ik}{\tilde{y}}_{ikl}\{-\Sigma_{kl}^{-1}\left(\mu_{kl}-x_{i}\right)\\ & & +\Sigma_{kl}^{-1}\frac{\sum_{m=1}^{M}\left(\mu_{kl}-s_{mkl}\right)\tilde{H}\left(s_{mkl}|\Omega_{k}\right)}{\sum_{m=1}^{M}\tilde{H}\left(s_{mkl}|\Omega_{k}\right)}\}. \end{array}$$ (43)

The solution ∂*E*(Π, Θ|*X*)/∂*μ*_*kl*_ = 0 yields the updating function for *μ*_*kl*_ during the iterations:

$$\begin{array}{c} \mu_{kl}=\frac{\sum_{i=1}^{N}{\tilde{z}}_{ik}{\tilde{y}}_{ikl}x_{i}}{\sum_{i=1}^{N}{\tilde{z}}_{ik}{\tilde{y}}_{ikl}}-\frac{\sum_{m=1}^{M}\left(\mu_{kl}-s_{mkl}\right)\tilde{H}\left(s_{mkl}|\Omega_{k}\right)}{\sum_{m=1}^{M}\tilde{H}\left(s_{mkl}|\Omega_{k}\right)}. \end{array}$$ (44)

### Covariance matrix estimation

Considering the derivation of function *E*(Π, Θ|*X*) in Eq (32) with respect to Σ_*kl*_, we have

$$\begin{array}{ccc} \frac{\partial E(\Pi ,\Theta |X)}{\partial \Sigma_{kl}} & = & -\sum_{i=1}^{N}{\tilde{z}}_{ik}{\tilde{y}}_{ikl}\{\frac{1}{2}\Sigma_{kl}-\frac{1}{2}\left(x_{i}-\mu_{kl}\right){\left(x_{i}-\mu_{kl}\right)}^{T}\\ & & -\frac{\int_{\Omega_{k}}\Phi \left(x|\mu_{kl},\Sigma_{kl}\right)\tilde{H}\left(x|\Omega_{k}\right)(\frac{1}{2}\Sigma_{kl}-\frac{1}{2}\left(x_{i}-\mu_{kl}\right){\left(x_{i}-\mu_{kl}\right)}^{T})dx}{\int_{\Omega_{k}}\Phi \left(x|\mu_{kl},\Sigma_{kl}\right)\tilde{H}\left(x|\Omega_{k}\right)dx}\}. \end{array}$$ (45)

Similar to Eq (41), the term $\int_{\Omega_{k}}\Phi \left(x|\mu_{kl},\Sigma_{kl}\right)\tilde{H}\left(x|\Omega_{k}\right)(\frac{1}{2}\Sigma_{kl}-\frac{1}{2}\left(x_{i}-\mu_{kl}\right){\left(x_{i}-\mu_{kl}\right)}^{T})dx$ in the Eq (45) can be approximated as:

$$\begin{array}{ccc} & & \int_{\Omega_{k}}\Phi \left(x|\mu_{kl},\Sigma_{kl}\right)\tilde{H}\left(x|\Omega_{k}\right)(\frac{1}{2}\Sigma_{kl}-\frac{1}{2}\left(x_{i}-\mu_{kl}\right){\left(x_{i}-\mu_{kl}\right)}^{T})dx\\ & & \;\approx \frac{1}{2M}\sum_{m=1}^{M}(\Sigma_{kl}-\left(s_{mkl}-\mu_{kl}\right){\left(s_{mkl}-\mu_{kl}\right)}^{T})\tilde{H}\left(s_{mkl}|\Omega_{k}\right). \end{array}$$ (46)

By setting ∂*E*(Π, Θ|*X*)/∂Σ_*kl*_ = 0, we obtain the updating function for Σ_*kl*_ during the iterations:

$$\begin{array}{ccc} \Sigma_{kl} & = & \frac{\sum_{i=1}^{N}{\tilde{z}}_{ik}{\tilde{y}}_{ikl}\left(x_{i}-\mu_{kl}\right){\left(x_{i}-\mu_{kl}\right)}^{T}}{\sum_{i=1}^{N}{\tilde{z}}_{ik}{\tilde{y}}_{ikl}}\\ & & -\frac{\sum_{m=1}^{M}\left(\left(s_{mkl}-\mu_{kl}\right){\left(s_{mkl}-\mu_{kl}\right)}^{T}-\Sigma_{kl}\right)\tilde{H}\left(s_{mkl}|\Omega_{k}\right)}{\sum_{m=1}^{M}\tilde{H}\left(s_{mkl}|\Omega_{k}\right)}. \end{array}$$ (47)

### Prior probability estimation

Because the prior distribution of between-cluster *π*_*ik*_ satisfies constraint $\sum_{k=1}^{K}\pi_{ik}=1$, Lagrange’s multiplier *τ*_*i*_ is used to enforce these constraints for each data point:

$$\begin{array}{c} \frac{\partial }{\partial \pi_{ik}}[E-\sum_{i=1}^{N}\tau_{i}(\sum_{k=1}^{K}\pi_{ik}-1)]=0. \end{array}$$ (48)

Constraint $\sum_{k=1}^{K}\pi_{ik}=1$ allows:

$$\begin{array}{c} \pi_{ik}=\frac{{\tilde{z}}_{ik}+E_{ik}}{\sum_{h=1}^{K}\left({\tilde{z}}_{ih}+E_{ih}\right)}. \end{array}$$ (49)

Similarly, we obtain the updating function for the prior distribution of within-cluster *η*_*ikl*_ under constraint $\sum_{l=1}^{L}\eta_{ikl}=1$:

$$\begin{array}{c} \eta_{ikl}=\frac{{\tilde{y}}_{ikl}+F_{ikl}}{\sum_{h=1}^{L}\left({\tilde{y}}_{ikh}+F_{ikh}\right)}. \end{array}$$ (50)
